# The Micromorphology and Histochemistry of Foliar Mixed Indumentum of *Leucas lavandulaefolia* (Lamiaceae)

**DOI:** 10.3390/plants10091767

**Published:** 2021-08-25

**Authors:** Yougasphree Naidoo, Thobekile Dladla, Yaser Hassan Dewir, Serisha Gangaram, Clarissa Marcelle Naidoo, Hail Z. Rihan

**Affiliations:** 1 School of Life Sciences, University of KwaZulu-Natal, Westville, Private Bag X54001, Durban 4000, South Africa; naidooy1@ukzn.ac.za (Y.N.); tdladlaza@yahoo.com (T.D.); serishagangaram@yahoo.com (S.G.); naidooclarissa5@gmail.com (C.M.N.); 2 Plant Production Department, College of Food and Agriculture Sciences, King Saud University, P.O. Box 2460, Riyadh 11451, Saudi Arabia; 3 Faculty of Science and Environment, School of Biological Sciences, University of Plymouth, Drake Circus PL4 8AA, UK; hail.rihan@plymouth.ac.uk; 4 Phytome Life Sciences, Launceston PL15 7AB, UK

**Keywords:** bicellular non-glandular trichomes, capitate trichomes, medicinal plants, peltate trichomes, secretion mode

## Abstract

*Leucas lavandulaefolia* Sm. (Lamiaceae) is an important medicinal plant with a broad spectrum of pharmacological activities. This study aimed at characterizing the morphology, distribution, and chemical composition of the secretions of trichomes at different developmental stages on the leaves of *L. lavandulaefolia*, using light and electron microscopy. Morphological observations revealed the presence of bicellular non-glandular, glandular peltate, and capitate trichomes on both adaxial and abaxial leaf surfaces. The density of both non-glandular and glandular trichomes decreased with the progression of leaf development. Heads of peltate and short-stalked capitate trichomes were between 20.78–42.80 µm and 14.98–18.93 µm at different developmental stages. Furthermore, long-stalked capitate trichomes were rare and infrequent. Leaf sections revealed the presence of important secondary metabolites in glandular trichomes, i.e., terpenoids. This study represents the first report on the morphology and histochemistry of trichomes of *L. lavandulaefolia*; therefore, there is a great scope for further research in this field.

## 1. Introduction

The genus *Leucas* R. Br. is one of the largest genera belonging to the family Lamiaceae and subfamily Lamioideae [1]. Currently, around 100 *Leucas* species have been recognized and distributed across the continent, ranging from southern to tropical Africa, throughout the Indian subcontinent, and subtropical parts of Asia, Australia, and Arabia [1,2]. The highest species diversity and richness for the genus *Leucas* has been identified in East Africa [3,4]. Likewise, *L. lavandulaefolia* Sm. (Syn. *L. linifolia*) displays a similar distribution pattern, and thrives in variable habitats, including wetlands, grasslands, rocky hills, jungles, and disturbed areas such as roadsides [5].

In traditional medicine, various parts of the plant are often used to combat several illnesses, such as the common cold, high fever, skin disease, headache, and conjunctivitis [6,7,8]. These uses are possibly affiliated with the presence of terpenoids, which are detected in many *Leucas* species such as *L. zeylanica* and *L. cephalotes*, which are well known for being rich in medicinal value [8,9,10]. Furthermore, despite the general usage of *L. lavandulaefolia*, studies have reported a range of biological activities related to this species, including antimicrobial, hepatoprotective, hypoglycemic, anti-inflammatory, antitussive, anthelmintic, febrifuge, and wound-healing properties [7,8,9,10].

Important medicinal compounds are located in microscopy plant structures identified as trichomes [11,12,13,14]. Trichomes are either unicellular or multicellular appendages that originate from epidermal cells, and vary in their morphology, location, and secretory ability [8]. Even though the morphologies of trichomes differ significantly, these structures can be grouped into two types: glandular (secreting) and non-glandular (non-secreting) trichomes [15,16]. Trichome diversity is expected to be of significance in taxonomic studies for intrageneric and intergeneric taxon identification in Lamiaceae [17] and for intrageneric taxon identification in genus *Leucas* [18].

Despite the potential ethnomedical uses and pharmacological value of *L. lavandulaefolia*, very little information is available on leaf morphology and histochemistry. Therefore, this study aimed to investigate the distribution, micromorphology, and chemical composition of the secretions of trichomes present on *L. lavandulaefolia* leaves, at three developmental stages, using light and electron microscopy. Information from this study will provide baseline information on the possible usage of the leaf extracts from *L. lavandulaefolia*.

## 2. Materials and Methods

### 2.1. Plant Material Collection

Leaf samples from *L. lavandulaefolia* were collected from 22 Abergeni Crescent, Durban North, KwaZulu-Natal (29°48′ S, 31°00′ E), at a moderate hill slope (55 m elevation). The collected samples were identified by a plant taxonomist, verified by Prof. A. Nicholas, and a voucher specimen (Dladla 1) was deposited in the Ward Herbarium of the University of KwaZulu-Natal, Westville Campus. Three leaf samples of *L. lavandulaefolia*, from the first, second, and third internode, for the emergent, young, and mature developmental stages, were used in this study.

### 2.2. Scanning Electron Microscopy

Fresh leaf segments were sectioned from the midrib and margin regions (approximate area: 2–5 mm^2^) and were rapidly quenched in liquid nitrogen (LN) and freeze-dried for 96 h in an Edward Modulyo freeze-dryer (Edward, Sussex, UK) at −40 to −60 °C in a vacuum (10^−2^ Torr). The leaf segments were secured onto brass stubs with double-sided carbon conductive tape, and sputter-coated with gold for 3 min, using a Polaron SC500 Sputter Coater (Hemel Hempstead, London). A LEO 1450 scanning electron microscope at 5 kV was used to observe the leaf samples [19]

### 2.3. Light and Transmission Electron Microscopy

Leaves of *L. lavandulaefolia* were cut into segments of area 1–2 mm^2^, fixed overnight using 2.5% glutaraldehyde, and buffered with 0.1 M phosphate at pH 7.2 with 0.5% caffeine. Specimens were post-fixed with 0.5% osmium tetroxide for 1.5 h [20]. After post-fixation, the samples were serially dehydrated with acetone, 5 min each at 30, 50, and 75%, and two sessions for 10 min at 100%. Following acetone dehydration, the samples were progressively infiltrated overnight using equal parts (50:50) of 100% acetone and Spurr resin [21]. After infiltration for 8 h, the specimens were placed in 100% Spurr resin in an oven at 70 °C for polymerization. Semi-thin sections, ranging in thickness from 0.5–2 µm, were then made with glass knives, using Ultracut-E ultramicrotome (Reichert-Jung, Austria), and fixed onto pre-cleaned glass. Ultrathin sections of 80–90 nm were picked up on uncoated 200-square-mesh copper grids and stained for 10 min with 2.5% uranyl acetate at 23 °C, washed with distilled water, and thereafter further stained for 10 min with lead citrate. Copper grids were further washed with distilled water and imaged using the JEOL 1010 TEM (JEOL, Tokyo, Japan) equipped with the iTEM software. Semi-thin (0.5–2 µm) and ultra-thin sections of leaf material of approximately 110 nm were prepared also for light microscopy. These sections were then mounted onto glass slides, stained with 0.05% aqueous solution of toluidine blue-O dissolved in 0.1% sodium carbonate at pH 11.1 [22], and observed using an Eclipse 80i light microscope (Nikon, Japan) [23].

### 2.4. Histochemistry

Histochemical tests are useful for studying the major components of the secretory products of trichomes. Histochemical analyses were performed on free-hand sections of fresh leaf tissue to detect the localization and presence of target chemical constituents within the leaves of *L. lavandulaefolia*, and were stained by the following reagents: (a) NADI reagent (0.1% α-naphthol + 1% *N*,*N*-dimethyl-p-phenylenediamine) for essential oils—samples were placed in the reagent for 1 h in the dark, washed with a 0.1 M sodium phosphate buffer, mounted, and viewed [24,25]; (b) 1% Nile blue for acidic and neutral lipids—sections were immersed in the stain for 5 min, transferred to 1% acetic acid for 1 min, mounted, and viewed [25,26]; (c) 2% Wagner and Dittmar reagents for alkaloids—sections were stained for 10 min each with both Wagner and Dittmar reagents, rinsed with distilled water, mounted, and viewed [25,27,28]; (d) 1% ruthenium red for mucilage and pectin—leaf sections were placed in the solution for 5 min, rinsed twice in distilled water, mounted, and viewed [25,27]; (e) 10% ferric trichloride for phenolic compounds—sections were immersed in the stain, a drop of sodium carbonate was added, and a positive reaction was obtained after 15 min [25]; (f) Sudan black B—sections were stained for 30 min, replaced with 70% ethanol, rinsed with distilled water, and mounted [25]. Control tests were carried out for all the histochemical methods, according to the suggestions of the respective authors. A Nikon Eclipse 80i Light Microscope (Nikon, Japan) was used to observe all the treated samples and capture their images. 

## 3. Results and Discussion

### 3.1. Microscopic Characterization of Foliar Secretory Structures

**Figure 1:**
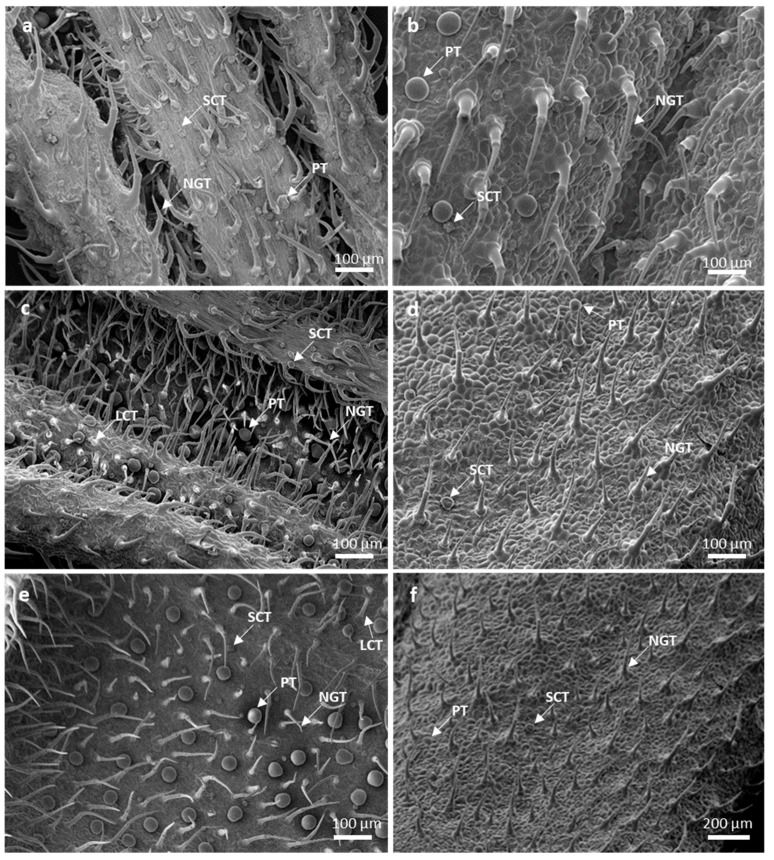
Scanning electron micrographs of trichomes found across three leaf developmental stages in *Leucas lavandulaefolia*, (**a**) emergent abaxial surface (midvein); (**b**) emergent adaxial surface (margin); (**c**) young abaxial surface (midvein); (**d**) young adaxial surface (margin); (**e**) mature abaxial surface (midvein); (**f**) mature adxial surface (margin). Abbreviations: PT—peltate trichome; SCT—short-stalked capitate trichome; LCT—long-stalked capitate trichome; NGT—non-glandular trichome.

The leaves of *L. lavandulaefolia* possess non-glandular and glandular trichomes on both adaxial and abaxial surfaces (Figure 1a–f). The density of these trichomes varies according to the leaf developmental stage. Non-glandular trichomes occur on both leaf surfaces of emergent and young leaves, with dense distribution on the abaxial leaf surface along the leaf veins and margins (Figure 1a,c). The frequency of fully developed bicellular non-glandular trichomes is higher on the emergent leaves curved in the leaf apex direction. These trichomes form a dense coverage over the glandular trichomes and shield them via this orientation. This orientation is used to cover and protect glandular trichomes, particularly during the early stages of leaf development [29,30]. In various species within *Leucas*, such as *L. decemdentata*, *L. chinensis*, *L. lanata*, and *L. helianthemifolia*, the non-glandular trichomes were generally found more on the abaxial than the adaxial leaf surface [5]. The density of both non-glandular and peltate trichomes decreased with the progression of leaf development (Figure 1e,f). According to Werker [9] and Fahn [10], cells of leaves may increase during growth, therefore allowing the amount of trichomes to decrease as surface area increases. It is assumed that, at the mature leaf stages, when the epidermis is fully developed, the role of trichomes become unimportant; therefore, they usually shed and senesce [9].

Three types of glandular trichomes are observed on *L. lavandulaefolia* leaf surface: peltate and short- and long-stalked capitate trichomes. Peltate trichomes are distributed on both leaf surfaces, with a dense distribution on the abaxial leaf surface throughout the emergent (Figure 1a), young (Figure 1c), and mature stages (Figure 1e). Glandular trichomes of *L. lavandulaefolia* undergo rapid differentiation during early leaf development. This provides a protective barrier on emergent leaves, which are highly susceptible to damage by external pressure [31,32,33]. The secretory stages of short- and long-stalked capitate trichomes are observed on the emergent young and mature leaves; however, their distribution is scarce. Short-stalked capitate trichomes occur on both leaf surfaces in between the peltate trichomes, with a dense distribution on the adaxial leaf surface (Figure 1a–f). These trichomes are observed on both surfaces of mature leaves (Figure 1e,f).

**Figure 2:**
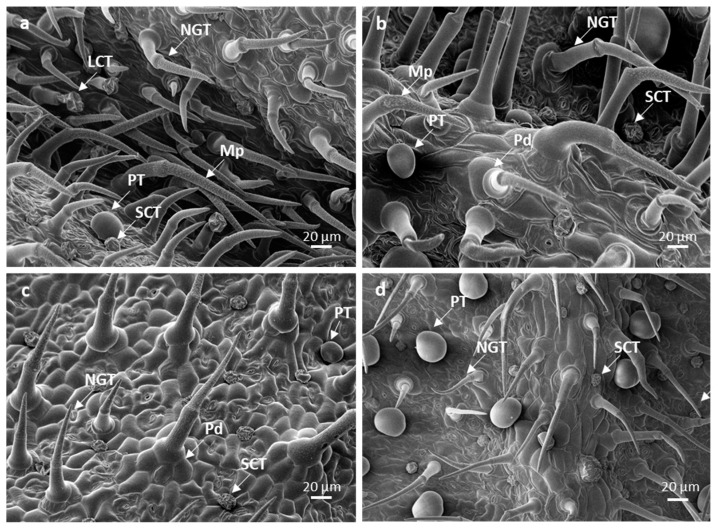
Scanning electron micrographs of trichomes found on the leaves of *Leucas lavandulaefolia*. (**a**,**b**) Glandular trichomes found between the dense coverage of non-glandular trichomes; (**c**,**d**) shorted-stalked and peltate glandular trichomes found between the non-glandular trichomes. Abbreviations: NGT—non-glandular trichome; PT—peltate trichome; SCT—short-stalked capitate trichome; LCT—long-stalked capitate trichome; Mp—micropapillae; Pd—pedestal.

**Figure 3:**
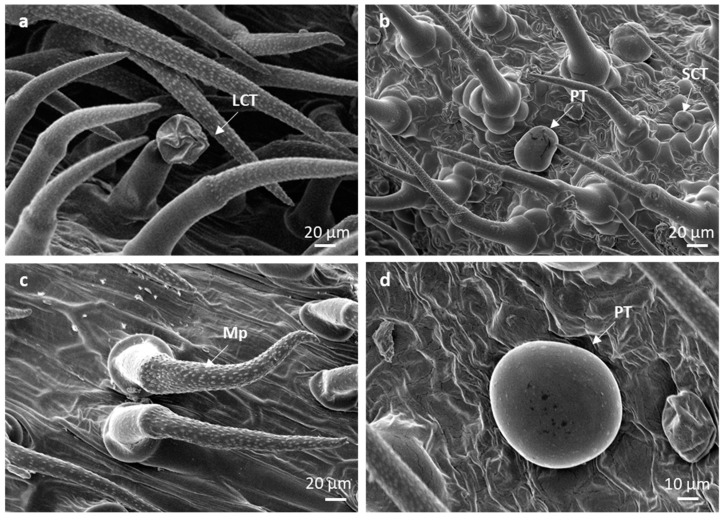
Scanning electron micrographs of different trichome types found on leaves of *Leucas lavandulaefolia*. (**a**) Long-stalked capitate; (**b**) peltate and short-stalked glandular trichomes; (**c**) micropapillae noted on the stalks of the non-glandular trichomes; (**d**) peltate trichome. Abbreviations: PT—peltate trichome; SCT—short-stalked capitate trichome; LCT—long-stalked capitate trichome; Mp—micropapillae.

Non-glandular trichomes are bent and lean slightly towards the leaf apex, forming a dense coverage over the glandular trichomes (Figure 2a,b). The non-glandular trichomes are supported by a cellular pedestal, formed by four–six epidermal cells arranged in a circle around the base (Figure 2a–d), perhaps providing support that anchors the trichome to cover and shield the surrounding glandular trichomes. It has been reported that the basal cellular pedestal provides both mechanical support and a strong point of attachment between the trichomes and the epidermal layer [34]. The surface of these trichomes is densely covered by numerous micropapillae, as wart-like outgrowths in all three leaf developmental stages (Figure 2a,b and Figure 3a,c). The non-glandular trichomes of Lamiaceae species are characterized by warty surfaces [35]. These structures are believed to arise from the cuticle, indicating maturity [8], and thicken the trichome walls, contributing to their function. The cuticular micro-ornamentations serve as a water-repellent surface, useful for plant self-cleaning [36,37]. Therefore, this indicates that the non-glandular trichomes on *L. lavandulaefolia* leaves mature at early stages of development. Short and long bicellular non-glandular trichomes were also observed on the stems or leaves of other *Leucas* species: *L. angularis*, *L. aspera*, *L. chinensis*, *L. ciliata*, *L. hirta*, *L. linifolia*, *L. martinicensis*, *L. vestita*, and *L. zeylanica* [18,38]. 

**Figure 4:**
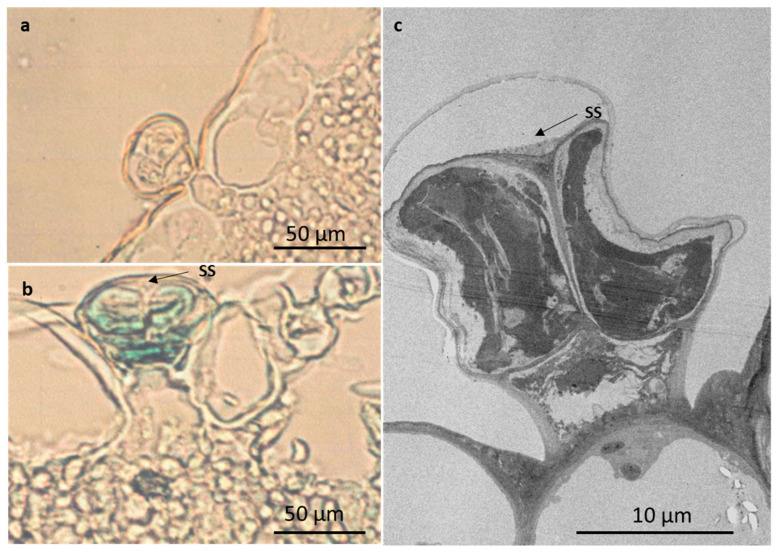
Light and electron micrographs of glandular trichomes found on the leaf surface of *Leucas lavandulaefolia*. (**a**) Short-stalked capitate trichome; (**b**,**c**) peltate trichome. Abbreviation: SS—subcuticular space.

Peltate trichomes consist of a basal epidermal cell, a subsessile stalk cell, and a large round head with four, or rarely, five cells (Figure 2a–d and Figure 3b,d). Peltate trichomes with four head cells have been recorded in some Lamioideae species [35,36]; however, this varies from four to 16 cells, depending on the species [39]. A few peltate glandular trichomes were observed on the leaves and stems of *L. cephalotes* Spreng [40], whilst Dwivedi and Joshi [41] reported four-celled glandular peltate hairs on the ovular surface of *L. urticifolia* Sm. Mature peltate trichomes are sunken in shallow epidermal depression (Figure 2d). The secretory head of these trichomes ranges from 20.78–3.89 µm on emergent, 30.66–3.00 µm on young, and 42.80 ± 2.82 µm on mature leaves (Table 1). Secretory mechanisms within specialized secretory structures such as trichomes (glandular) consist of three main processes: the early or pre-secretory stage, the secretory stage, and the post-secretory stage [42,43,44]. The pre-secretory stage is often identified by the continuous differentiation of a trichome up until the formation of a fully developed structure [42,45]. Mature leaves show high numbers of peltate trichomes with a smooth balloon or slightly flattened head at the secretory stages (Figure 2d). There is a ruptured line of weakness from the central part of the head cuticle to the base for secretory release (Figure 3b). The head cells of peltate trichomes are covered by a large subcuticular space, onto which the secreted material accumulates (Figure 4b,c). During the secretory stage, there is a strong accumulation of secretions such as oils in the subcuticular space [45]. Interestingly, the major difference often observed between the pre-secretory and the secretory stage is the amount of secretion [42,43]. 

Short-stalked capitate trichomes consist of a basal epidermal cell, a subsessile stalk cell, and a head with four cells (Figure 2a–d and Figure 4a). The head cells of these trichomes varied from 14.98 ± 3.29 µm on emergent, 17.12 ± 0.74 µm on young, and 18.93 ± 4.96 µm on mature leaves (Table 1). Long-stalked capitate trichomes are rare and infrequent on the abaxial leaf veins or adaxial leaf grooves, in between the dense coverage of non-glandular trichomes (Figure 2a). These trichomes consist of a basal cellular pedestal of six to eight epidermal cells, a long stalk of one or two cells, and a head of four cells (Figure 3a). Long-stalked capitate trichomes were hardly observed at each developmental stage, therefore it was an impossible task to locate and measure the diameter of the head. The head cells of both short- and long-stalked trichomes appeared “ruptured” (Figure 2a). Post-secretory release occurs by rupture of the head cuticle along the lines of weakness opposite the fusion sutures in between the head cells. These sutures rupture from the central part of the head cuticle to the base. Subsequently, before the rupture of the cuticle, the exudation of secretions directly increases the area within the subcuticular space [44,45]. The post-secretory stage is indicated by visible secretions into the external environment, followed by a prominent collapse of the subcuticular space [44,45]. Bicellular non-glandular, peltate, and short- and long-stalked capitate trichomes are observed on both surfaces of *L. lavandulaefolia* leaves, with a higher abundance of non-glandular trichomes, especially on the abaxial leaf veins and apex.

### 3.2. Histochemistry

Histochemical analyses are valuable tools which detect and locate essential metabolites present in plant tissues or secretions. Results of the histochemical tests for glandular and non-glandular trichomes are presented in Table 2. Essential oils were detected using the NADI reagent, while Nile blue A was used to test for acidic lipids. The presence of alkaloids was tested using Ditmar and Wagner reagents. Total phenolics and pectin was tested using ferric trichloride and ruthenium red, respectively. Additionally, Sudan black B detected lipids and cutinized or suberized cell walls. 

**Figure 5:**
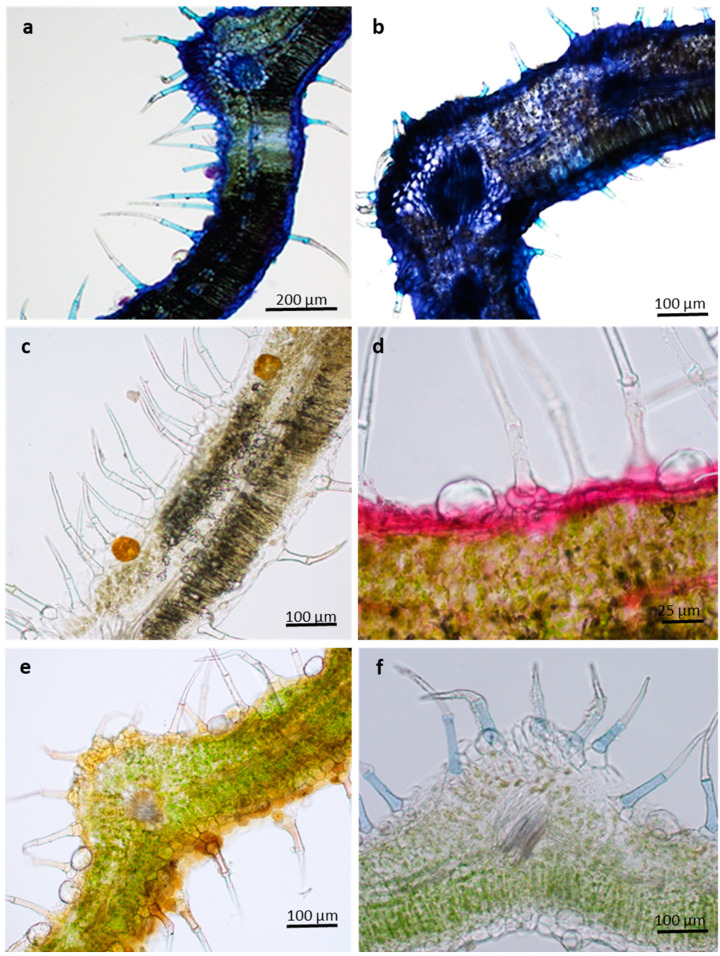
Histochemical characterization of *L. lavandulaefolia* leaves. (**a**) Essential oils stained purple; (**b**) negative staining of acidic lipids; (**c**) alkaloids in leaf tissue stained orange-brown; (**d**) mucilage and pectin stained red-pink in leaf tissue; (**e**) phenols stained brown (**f**) lipids stained blue.

Glandular trichomes are regarded as primary secretory organs, secreting primary and secondary metabolites that are widely used in pharmaceuticals and fragrances. Their secretions play a major role in plant–animal interactions, acting as protectors against insects, microbes, and herbivores [46]. Capitate trichomes of *L. lavandulaefolia* contain small amounts of essential oils, phenolic compounds, and mucilage in their small subcuticular spaces. These viscous secretions are speculated to exhibit antiherbivory action, such as food deterrents or insect repellents [47,48]. The majority of the essential oils are produced by the large peltate glandular trichomes. These trichomes stained purple, with strong reactions in head cells and subcuticular spaces. This indicates the presence of a combination of low and high molecular weight terpenoids (Figure 5a). However, the blue coloration observed in this figure is merely of the hydrophobic walls as a result of cutinization (epidermis) or lignification (xylem).

Acidic lipids were not detected in the leaf tissue and base of non-glandular trichomes (Figure 5b). Alkaloids were absent in the trichomes; however, iodine from the Wagner and Dittmar reagents stained starch grains black throughout the leaf tissue, as in Figure 5c. Glandular trichomes are rarely the ideal structures for the alkaloid storage, ensuring rapid release of these compounds in the event of an attack [49]. When both alkaloids and essential oils are secreted, it is considered a double defense mechanism against herbivory, because they serve the same function of deterring herbivory [50]. The terpenes and alkaloids have been reported to possess antimicrobial and antiherbivore properties [47,51]. 

Mucilage and pectin were not detected in both glandular and non-glandular trichomes (Figure 5d); phenolic compounds are only slightly present only in the head cells of the peltate trichomes and absent in the non-glandular trichomes (Figure 5e). Lipids are usually detected in the peltate glandular trichomes; however, these trichomes exhibited a negative result, potentially due to the highly impermeable cuticle of peltate trichomes found in Lamiaceae. Non-secretory lipids, however, were detected in the cellular pedestal and basal cells of the non-glandular trichomes (Figure 5f). These compounds play a supportive or protective role in the plant–environment interactions [46]. It is assumed that non-glandular trichomes play an important role in leaf protection by reflecting solar radiation and incoming UV light on the leaf surface, to prevent excessive water loss [39]. This, in turn, helps the plant to maintain a constant leaf temperature [52].

Various types of glandular trichomes observed in species within the genus *Leucas* can be regarded as phylogenetically useful and taxonomically valuable [5]. The combination of several microscopic techniques aided in the identification of various glandular and non-glandular trichomes. This study showed that the secretions of peltate and capitate trichomes release gradually by the rupture of head cuticles in between the head cells. This secretion mode confirms the mode observed in *Ocimum obovatum* [29], *Plectranthus zuluensis* [30], *Leonotis leonurus* [33], and *Plectranthus ornatus* [34].

## 4. Conclusions

The present study will represent the first detailed micromorphological investigation of the trichomes in *L. lavandulaefolia*. The structural investigations of this study provide additional information on the distribution, micromorphology, and secretion mode of the foliar trichomes of *Leucas* genus. The distribution and type of trichomes found in *L. lavandulaefolia* can be considered a useful diagnostic tool for taxonomists and may show a systematic relationship among species. Non-glandular and glandular (peltate and capitate) trichomes were found across all leaf developmental stages. However, the density of both types of trichomes decreased from the emergent to the mature leaf stages. In addition, histochemical tests indicated the presence of various compounds with possible medicinal importance. This information is vital, as it will improve our knowledge of the role of these medicinal compounds in the plant’s ecology. Complete phytochemical analyses are needed to further identify the constituent compounds in these trichomes with potential medicinal properties. To the best of our knowledge, this research will be the first report on the morphology and histochemistry of trichomes of *L. lavandulaefolia*; therefore, there is a great scope for further research in this field.

## Figures and Tables

**Table 1:** Glandular trichome head measurements (diameter) across three leaf developmental stages of *L. lavandulaefolia*.

	Leaf Developmental Stage		
Trichome Type	Emergent	Young	Mature
Peltate	20.78 ± 3.89 µm	30.66 ± 3.00 µm	42.80 ± 2.82 µm
Short-stalked	14.98 ± 3.29 µm	17.12 ± 0.74 µm	18.93 ± 4.96 µm

**Table 2:** Histochemical identification of target chemical constituents of glandular trichomes on *Leucas lavandulaefolia* leaves. Results: (−) negative reaction; (+) moderate reaction; (++) strong reaction.

Target Constituents	Test	Peltate Trichomes		Capitate Trichomes		Non-Glandular Trichomes		Colour Reaction
		Head Cells	Subcuticular Space	Head Cells	Subcuticular Space	Cellular Pedestal	Basal Cells	
Essential oils	NADI reagent	++	++	+	+	−	−	Positive—blue
Acidic lipids	Nile blue A	−	−	−	−	−	−	Positive—blue
Alkaloids	Wagner and Dittmar	−	−	−	−	−	−	Positive—orange/brown
Phenolic compounds	Ferric trichloride	+	−	+	+	−	−	Positive—black deposits
Mucilage and pectins	Ruthenium red	−	−	−	−	−		Positive—pink/red
Lipids	Sudan Black	−	−	−	−	++	++	Positive—blue-black

## Data Availability

Data is contained within the article.
